# 221. Effectiveness of RSV Vaccines in Older Adults in the United States, VISION Network, 2023-2025

**DOI:** 10.1093/ofid/ofaf695.079

**Published:** 2026-01-11

**Authors:** Ruth Link-Gelles, Patrick K Mitchell, Janet Watts, Toan Ong, Sarah W Ball, Malini B DeSilva, Kristin K Dascomb, Stephanie Irving, Shaun J Grannis, Nicola P Klein, Michelle Barron, David Mayer, Catia Chavez, Angela Cheung, Lawrence Reichle, Charlene E McEvoy, Omobosola Akinsete, Jingran Cao, Tamara Sheffield, Daniel Bride, Julie Arndorfer, Joshua Van Otterloo, Allison L Naleway, Ousseny Zerbo, John R Hansen, Lawrence Block, Karen B Jacobson, Colin Rogerson, Thomas Duszynski, William F Fadel, Karthik Natarajan, Josephine Mak, Morgan Najdowski, Amber Kautz, Ryan E Wiegand, Allison Avrich Ciesla, Jennifer DeCuir, Amanda B Payne

**Affiliations:** Centers for Disease Control and Prevention, Atlanta, Georgia; Westat, Rockville, Maryland; Westat, Rockville, Maryland; University of Colorado Anschutz Medical Campus, Centennial, Colorado; Westat, Rockville, Maryland; HealthPartners Institute, Bloomington, Minnesota; Intermountain Healthcare, Murray, Utah; Kaiser Permanente Center for Health Research, Portland, Oregon; Indiana University, Indianapolis, Indiana; Division of Research Kaiser Permanente Vaccine Study Center, Oakland, California; University of Colorado, Aurora, Colorado; University of Colorado Anschutz Medical Campus, Centennial, Colorado; University of Colorado, Aurora, Colorado; Westat, Rockville, Maryland; Westat, Rockville, Maryland; HealthPartners Institute, Bloomington, Minnesota; HealthPartners Institute, Bloomington, Minnesota; HealthPartners Institute, Bloomington, Minnesota; IntermountainHealth, Salt Lake City, Utah; Intermountain Healthcare, Murray, Utah; Intermountain Healthcare, Murray, Utah; IntermountainHealth, Salt Lake City, Utah; Kaiser Permanente Center for Health Research, Portland, Oregon; Division of Research Kaiser Permanente Vaccine Study Center, Oakland, California; Division of Research Kaiser Permanente Vaccine Study Center, Oakland, California; Kaiser Permanente Northern California, Oakland, California; Kaiser Permanente Vaccine Study Center, Oakland, California; Regenstrief Institute, Indianapolis, Indiana; Regenstrief Institute, Indianapolis, Indiana; Regenstrief Institute, Indianapolis, Indiana; Columbia University, New York, New York; Division of Healthcare Quality Promotion, Centers for Disease Control and Prevention, Atlanta, Georgia; CDC, Atlanta, Georgia; Centers for Disease Control and Prevention, Atlanta, Georgia; Centers for Disease Control and Prevention, Atlanta, Georgia; Centers for Disease Control and Prevention, Atlanta, Georgia; Centers for Disease Control and Prevention, Atlanta, Georgia; CDC, Atlanta, Georgia

## Abstract

**Background:**

Respiratory syncytial virus (RSV) caused approximately 100,000-160,000 hospitalizations annually in adults aged ≥60 years in the United States (US) before RSV vaccine introduction. In 2023, two vaccines were recommended for prevention of severe RSV disease in adults aged ≥60 years using shared clinical decision making. In 2024, a third product was licensed, and all three available vaccines were recommended for all adults aged ≥75 years and for adults aged 60-74 years at increased risk of severe RSV disease. We assessed post-licensure vaccine effectiveness (VE) to inform future recommendations and public communications.

**Figure:**
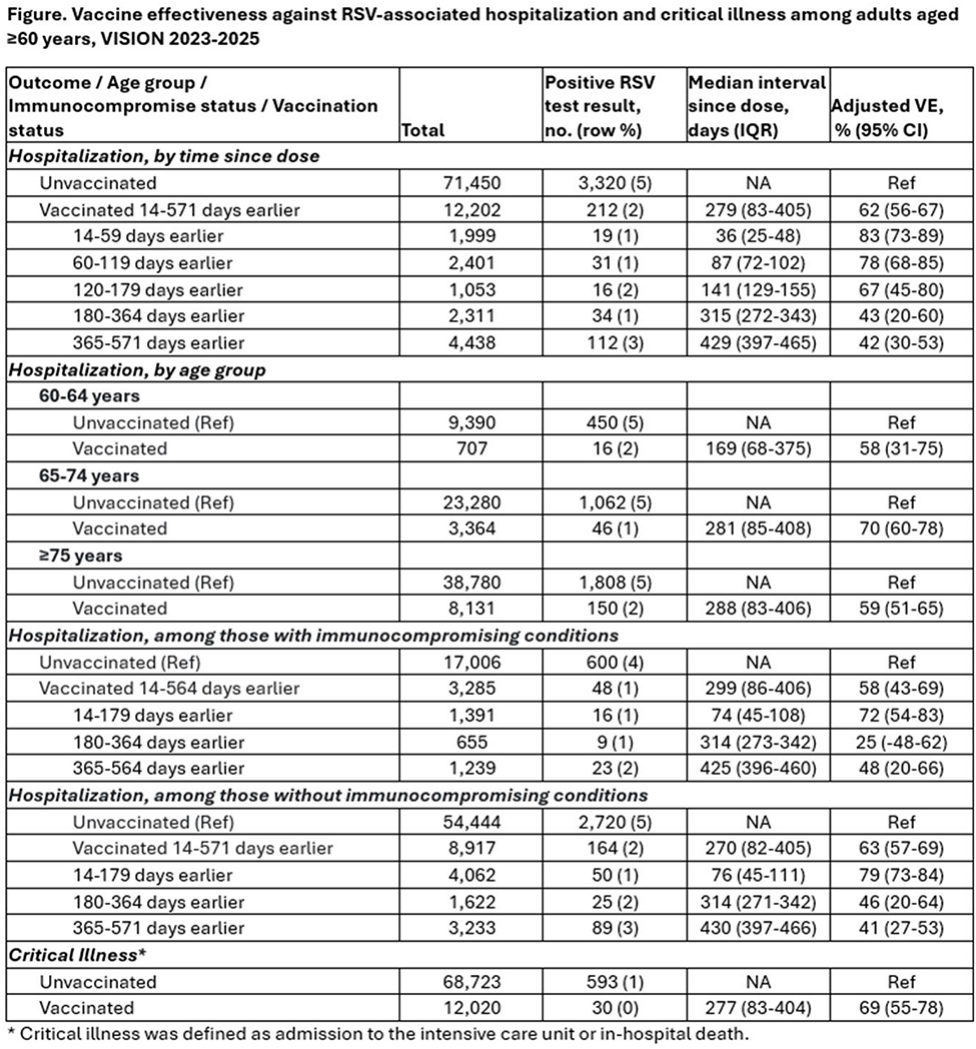

**Methods:**

VISION is a multi-site electronic health record-based study including >200 hospitals in the US. Adults aged ≥60 years hospitalized with RSV-like illness and tested for RSV were included. Cases had a positive molecular or antigen RSV test; controls had a negative molecular RSV test. Critical illness included admission to the intensive care unit or in-hospital death. VE against hospitalization and critical illness was calculated using a test negative design as (1-adjusted odds ratio) x 100% where the odds ratio compares odds of vaccination in cases and controls after adjusting for confounders. Results were stratified by time since RSV vaccination, age group, and immunocompromised status.

**Results:**

Among 83,652 hospitalizations during October 2023-March 2024 and October 2024-March 2025, VE was 62% (95% CI: 56-67%, Table) against RSV-associated hospitalization, median 279 days after RSV vaccination. VE was 83% (95% CI: 73-89%) at 14-59 days after vaccination and 42% (95% CI: 30-53%) at least 1 year after vaccination (median 429 days). VE against RSV-associated critical illness was 69% (95% CI: 55-78%), median 277 days after vaccination. VE was similar by age group and among those with and without immunocompromising conditions.

**Conclusion:**

RSV vaccines are effective at preventing severe RSV and have the potential to reduce the burden of RSV-associated hospitalizations among older adults, although waning protection was apparent during the second season after vaccination. Ongoing monitoring of RSV VE is warranted to ensure vaccines are working as expected, to understand duration of protection, and to inform policy decisions.

**Disclosures:**

All Authors: No reported disclosures

